# Not yet 90-90-90: A quality improvement approach to human immunodeficiency virus viral suppression in paediatric patients in the rural Eastern Cape, South Africa

**DOI:** 10.4102/safp.v62i1.5169

**Published:** 2020-10-15

**Authors:** James D. Porter, Mireille N.M. Porter, Maresa du Plessis

**Affiliations:** 1 Department of Family Medicine and Rural Health, Walter Sisulu University, Mthatha, South Africa; 2 Madwaleni Hospital, Elliotdale, South Africa

**Keywords:** quality improvement, task-shifting, paediatric HIV outcomes, clinical mentorship, viral suppression

## Abstract

**Background:**

A strategy implemented by the South African Department of Health to manage the high burden of human immunodeficiency virus (HIV) has been to task-shift services to primary health care clinics. Outcomes of paediatric patients with HIV are poorer than those of adults, particularly in rural areas. Viral suppression in paediatric patients at the feeder clinics of a rural South African hospital was anecdotally far below the aim of the Joint United Nations Programme on HIV/AIDS (UNAIDS) of 90%.

**Methods:**

A quality improvement approach was used to conduct a baseline assessment of HIV viral suppression in paediatric patients and other process measures, implement a clinical mentorship intervention and evaluate its effectiveness.

**Results:**

An initial audit of 235 clinical folders of paediatric patients with HIV revealed a viral suppression of 55.3%. Other poor measures included prescription accuracy, viral loads performed within schedule and response to successive high viral loads. A clinical mentorship intervention using dedicated doctor outreach was implemented and the audit repeated after 12 months (263 folders). Viral suppression improved to 67.4%, as did most other process measures.

**Conclusion:**

The quality improvement approach regarding the aim to significantly improve viral suppression in paediatric patients through the implementation of clinical mentorship was successful.

## Introduction

Madwaleni Hospital is a deeply rural district hospital on the ex-Transkei coast of the Eastern Cape, South Africa. The concern of clinicians about the number of paediatric admissions with virally unsuppressed human immunodeficiency virus (HIV) pointed to poor viral suppression rates at its primary health care (PHC) feeder clinics. Paediatric HIV care was previously hospital-based and managed by a local non-governmental organisation (NGO).^1^ A loss of funding for the NGO and very low doctor numbers in 2012^2^ contributed to a haphazard process of task-shifting where patient care was devolved to nine PHC clinics without doctor support. As of early 2016, unreliable TIER.Net^3^ data (because of a lack of trained data capturers at clinics) put the viral suppression in paediatric patients at roughly 50% for the Madwaleni feeder clinics.

According to UNAIDS data from 2018, 90% of the estimated 1.8 million HIV-positive children live in sub-Saharan Africa.^4^ With a view to end this epidemic by 2030, UNAIDS recommended the implementation of the ‘90-90-90’ targets: 90% of the population tested for HIV, 90% of those who are HIV positive on antiretroviral therapy (ART) and 90% viral suppression for those on ART.^5^ In an attempt to increase access to HIV care, the South African National Department of Health implemented the strategy of task-shifting, which moved the initiation of patients on ART and their subsequent management to nurse-run primary health clinics.^6^ Studies have subsequently found nurse-monitored HIV care of adult patients to be non-inferior to doctor-monitored HIV care in the South African context.^7,8^

Despite these global efforts, paediatric HIV care has not seen the same levels of success as adult HIV care.^9^ A 2016 meta-analysis showed levels of viral suppression in paediatric patients in resource-limited settings were 73%,^10^ compared with 84% in adults,^11^ with a 2019 study from Eswatini showing 78% viral suppression in paediatric patients.^12^ Rural children are particularly vulnerable with studies showing increased risk for poorer outcomes compared with urban counterparts,^13,14^ and poorer viral suppression with a 2017 study conducted in rural Limpopo showing between 48% and 52% viral suppression.^15^

To address the perceived poor HIV viral suppression in paediatric patients at the Madwaleni feeder clinics, a quality improvement (QI) approach was selected. The QI approach views health systems as a process with constant opportunity for improvement.^16^ It has the advantage of incorporating both research and action in the same process.^17,18^ Support for and training in QI methodology were received from the Stellenbosch University Collaborative Capacity Enhancement through Engagement with Districts (SUCCEED).^19^

The aim of this QI-driven research was therefore to improve the HIV viral suppression in paediatric patients at the Madwaleni PHC clinics from a presumed baseline of 50% to 90% within the space of 1 year (May 2016 to May 2017).

## Research methods and design

Madwaleni Hospital and its nine PHC clinics are in the Mbhashe subdistrict of the deeply rural Eastern Cape. According to TIER.Net data, an estimated 230 children were in care and on ART at the PHC clinics in 2016. All nine clinics had at least one professional nurse (PN) who had undergone the Nurse Initiated Management of Antiretroviral Treatment (NIMART) training. The national consolidated guidelines for the prevention of mother-to-child transmission of HIV (PMTCT) and the management of HIV in children, adolescents and adults^20^ came into effect from April 2015 and were used as the official guidelines for the duration of the QI cycle.

After using the data from TIER.Net to identify the problem of poor viral suppression in paediatric patients, a research or QI team was convened consisting of key stakeholders, including the acting clinical manager, acting CEO, family physician and family medicine registrar from Madwaleni Hospital, the operational managers of the PHC clinics, as well as a volunteer social worker and volunteer clinical researcher medical officer. The baseline file audit was planned for May 2016 with a gold standard of 90% viral suppression set, and various process measures discussed and decided upon: viral load (VL) performed within schedule, months after second high VL before regimen change, number of efavirenz (EFV) to lopinavir or ritonavir (LPV/r) changes, occurrence of stavudine (d4T) prescriptions, number of weights in last 12 months, correct dosing according to last recorded weight and daily versus twice-daily dosing of ART where applicable.

The baseline file audit date was set for 1st of May 2016. Data collection was performed using an Excel spreadsheet audit tool designed by the team. Inclusion criteria were all files where the child was less than the age of 15 years at the audit date and were considered ‘in care’, that is they had received ART at the PHC clinic within the 3 months preceding the audit date. No direct contact was made with the population for the purpose of data collection, and all data were collected from patient records only. To ensure confidentiality, patient names were removed during the data collection process and not recorded on the final data collection tool.

### Ethical consideration

Although this was a quality-improvement initiative, ethical clearance was still obtained from Human Research Committee, Faculty of Health Sciences postgraduate education, training Research and Ethics Unit, Walter Sisulu University (Clearance number: 033/2017). Site approval from the Madwaleni Hospital chief executive officer and provincial approval from the Eastern Cape Health Research Committee were also obtained.

## Baseline audit results

At the time of the baseline audit, 235 children were on ART and in care. Table 1 shows the demographic and baseline ART data. The median age was 9.1 years with 9.8% of children under the age of 4 years. There was an even gender split and a median age of ART initiation of 3.4 years. Just over half (52%) of patients were originally initiated on an EFV-based regimen.

**TABLE 1:** Demographic data and baseline antiretroviral therapy data.

Variables	2016	2017	*p*
**In care (N)**	235	263	
**Age (years)**			
Median	9.1	9.3	-
IQR	6.3–11.6	6.3–11.7	-
**Age (years)**			
0–3	-	-	0.8824
*N*	23	27	-
% (*n*/N)	9.8	10.3	-
4–10	-	-	0.5903
*N*	118	125	-
% (*n*/N)	50.2	47.5	-
11–15	-	-	0.6488
*N*	94	111	-
% (*n*/N)	40.0	42.2	-
**Gender**			
Female	-	-	1.0000
*N*	116	130	-
% (*n*/N)	49.4	49.4	-
Male	-	-	-
*N*	119	133	-
% (*n*/N)	50.6	50.6	-
**Age at ART initiation (years)**			
Median	3.4	3.2	-
IQR	1.0–6.5	1.0–6.0	-
**Age at ART initiation (years)**			
0–3	-	-	0.7193
*N*	109	127	-
% (*n*/N)	46.4	48.3	-
4–10	-	-	0.3675
*N*	112	114	-
% (*n*/N)	47.7	43.3	-
11–15	-	-	0.3865
*N*	14	22	-
% (*n*/N)	6.0	8.4	-
**Original ART regimen**			
ABC + 3TC + EFV	-	-	0.5655
*N*	73	89	-
% (*n*/N)	31.1	33.8	-
D4T + 3TC + EFV	-	-	0.2051
*n*	49	43	-
% (*n*/N)	20.9	16.3	-
TDF + FTC + EFV	-	-	1.0000
*n*	0	1	-
% (*n*/N)	0.0	0.4	-
ABC + 3TC + LPV/r	-	-	0.7017
*n*	74	88	-
% (*n*/N)	31.5	33.5	-
D4T + 3TC + LPV/r	-	-	0.5324
*n*	38	37	-
% (*n*/N)	16.2	14.1	-
AZT + 3TC + LPV/r	-	-	0.2204
*n*	1	5	-
% (*n*/N)	0.4	1.9	-
**Weight Z-score for age < 10 years (N)**	151	159	
< -3	-	-	1.0000
*n*	5	6	-
% (*n*/N)	3.3	3.8	-
≥ -3 < -2	-	-	1.0000
*N*	12	12	-
% (*n*/N)	7.9	7.5	-
≥ -2 < -1	-	-	0.4179
*N*	31	39	-
% (*n*/N)	20.5	24.5	-
≥ −1 < 0	-	-	1.0000
*N*	48	50	-
% (*n*/N)	31.8	31.4	-

ART, antiretroviral therapy; IQR, interquartile range; ABC, Abacavir; 3TC, lamivudine; EFV, efavirenz; D4T, stavudine; TDF, tenofovir; FTC, emtricitabine; AZT, zidovudine; LPV/r, lopinavir/ritonavir.

With regard to the current ART regimens shown in Table 2, none of the patients were on third-line ART. Abacavir (ABC), lamivudine (3TC) and zidovudine (AZT) were the most commonly used nucleoside reverse transcriptase inhibitors, with only three patients receiving tenofovir (TDF). It was of concern that 8.5% of patients were still on a d4T-containing regimen, whereas only 1.6% of patients who were originally taking an EFV-based regimen had been switched to an LPV/r-based regimen.

**TABLE 2:** The details of current antiretroviral therapy.

Variables	2016	2017	*p*
**In care (N)**	235	263	
**Current ART regimen**			
ABC + 3TC + EFV			
*n*	102	118	
% (*n*/N)	43.4	44.9	0.7189
D4T + 3TC + EFV			
*n*	8	0	
% (*n*/N)	3.4	0.0	0.0023
AZT+3TC+EFV			
*n*	5	0	
% (*n*/N)	2.1	0.0	0.0231
TDF + FTC + EFV			
*n*	3	4	
% (*n*/N)	1.3	1.5	1.0000
ABC + 3TC + LPV/r			
*n*	98	124	
% (*n*/N)	41.7	47.1	0.2409
ABC + AZT + LPV/r			
*n*	1	2	
% (*n*/N)	0.4	0.8	1.0000
D4T + 3TC + LPV/r			
*n*	12	1	
% (*n*/N)	5.1	0.4	0.0010
AZT + 3TC + LPV/r			
*n*	6	11	
% (*n*/N)	2.6	4.2	0.3363
3TC monotherapy			
*n*	0	2	
% (*n*/N)	0.0	0.8	0.5005
Any D4T-based regimen			
*n*	20	1	
% (*n*/N)	8.5	0.4	< 0.0001
**Original EFV-based regimen (N)**	122	133	
EFV changed to LPV/r			
*n*	2	11	
% (*n*/N)	1.6	8.3	0.0210

ART, antiretroviral therapy; IQR, interquartile range; ABC, Abacavir; 3TC, lamivudine; EFV, efavirenz; D4T, stavudine; TDF, tenofovir; FTC, emtricitabine; AZT, zidovudine; LPV/r, lopinavir/ritonavir.

Table 3 shows the data collected with regard to weights and dosing. Patients had weights recorded on an average of 4.4 times in the preceding 12 months, and a worrying 51.1% of patients had an incorrect prescription according to their last recorded weight (based on the weight-band dosing provided in the national guidelines).^20^ Only 18.1% of eligible patients were placed on daily ABC or 3TC dosing instead of twice-daily dosing. Daily dosing of ABC or 3TC is recommended by the national guidelines because of strong carer preference and a non-inferiority to twice-daily dosing in terms of viral suppression.^20,21^

**TABLE 3:** Weights and dosing.

Variables	2016	2017	*p*
**In care (N)**	235	263	
**Number of weights documented in last 12 months**			
Average	4.4	4.5	0.5664
**Months since last documented weight**			
Average	3.2	2.3	0.0269
**Dosing by last recorded weight**			
Any incorrect dose			
*n*	120	92	0.0004
% (*n*/N)	51.1	35.0	
Separate drug prescriptions (N)			
*n*	940	1052	
% (*n*/N)	-	-	
Overdosed			
*n*	67	61	
% (*n*/N)	7.1	5.8	0.0233
Underdosed			
*n*	110	67	
% (*n*/N)	11.7	6.4	< 0.0001
Incorrect frequency			
*n*	3	1	
% (*n*/N)	0.3	0.1	0.0337
**ABC/3TC dosing frequency >10 kg (N)**			
*n*	182	233	
% (*n*/N)	-	-	
Daily dosing			
*n*	33	103	
% (*n*/N)	18.1	44.2	< 0.0001

ABC, Abacavir; 3TC, lamivudine.

As can be seen in Table 4, of the 219 patients who were eligible for a VL testing (those who had been on their current ART regimen for 6 months or more), 72% had a recorded VL performed within the existing national guideline schedule (6 months after initiation of regimen change, then again after 12 months and annually thereafter).^20^ There was an average of 9.2 months’ wait before a repeat VL testing after a VL of more than 1000 RNA copies/mL, whereas the national guideline recommended a repeat after 2 months.^20^ Of the 219 eligible patients, only 121 (55.3%) had a suppressed VL. The definition of viral suppression used was a VL of less than 1000 RNA copies/mL performed within the last 13 months or performed within the last 7 months of ART initiation or a regimen change. In calculating viral suppression, patients who did not have their VL tested in the prescribed period were considered as having an unsuppressed VL. Viral suppression was slightly higher than the TIER.Net data of 50%, but still far below the UNAIDS goal of 90-90-90.

**TABLE 4:** Viral load data.

Variables	2016	2017	*p*
On ART and eligible for VL (N)	219	227	
Viral load timing			
Most recent VL performed within schedule†			
*n*	158	200	
% (*n*/N)	72.1	88.1	< 0.0001
Months between VLs if previous VL ≥ 1000			
Average			
*n*	9.2		-
% (*n*/N)	5.3		0.0011
Viral suppression			
< 1000 RNA copies/mL within schedule†			-
*n*	121	153	
% (*n*/N)	55.3	67.4	0.0088

VL, viral load; ART, antiretroviral therapy.

† , Viral load performed within last 13 months on ART or within 7 months from ART start or regimen change.

## Intervention methodology

The QI team made use of a combination of systematic team problem solving and benchmarking to develop an intervention to improve viral suppression in paediatric patients within the QI approach.^22^ In considering the cause of low viral suppression in paediatric patients, and looking at solutions that worked in similar settings, a clinical mentorship initiative was decided on. This was made possible by senior stability and an increased number of doctors employed at Madwaleni hospital from 2013.

A study in Khayelitsha (South Africa) had found that as a result of clinical mentorship ‘nurses improved their confidence in performing HIV related clinical tasks, nurses were initiating patients after mentorship and the quality of initiation and management was satisfactory’.^23^ A Botswana study looking at clinical mentorship for decentralised paediatric HIV care found that it may assist improvements in a number of important areas, including proper ART dosing and monitoring,^24^ whilst a QI study carried out in the North West Province of South Africa concluded that ‘task shifting in a paediatric ART programme in particular can be effective if patients continue to be closely monitored and PHC nurses are regularly supported by a PHC doctor’.^25^

From May 2016 a medical officer, community service medical officer or family medicine registrar was assigned a specific clinic which he or she visited on a monthly or fortnightly basis, depending on the total number of patients seen at the clinic per month. As far as possible, the same doctor did outreach to the same clinic, to promote continuity of care and assist in developing an effective clinical mentorship relationship with the clinic staff.^26^ The purpose of the doctor outreach was to assist by seeing complicated patients (down-referred from Madwaleni Hospital or booked by the clinic staff) together with the PNs. This was not limited to HIV-positive patients. Ongoing nurse training or education in the form of short sessions around different clinical topics were conducted on an ad hoc basis to strengthen the problem solving and clinical decision-making of the nurses,^27^ whilst increasing their confidence in managing ART.^23^ In addition to this, the doctors were asked to review a few folders having records of paediatric patients with HIV at each visit to monitor services and data.^28^

After 12 months, the audit was repeated using the same audit tool with the same inclusion criteria with a re-audit date of 1st May 2017. Data were captured and analysed using Microsoft Excel. Chi-squared test or Fisher exact test were used to test for association between categorical variables. Continuous data were tested for normality, and the appropriate statistical test was used to compare for significance of association between groups.

## Repeat audit results

As can be seen in Table 1, a further 28 patients were in care at the PHC clinics on the 1st of May 2017 compared with the 1st of May 2016, giving a total of 263 patients. The demographic data remained largely unchanged with a slightly lower median age of ART initiation of 3.2 years. There were no significant differences in the original ART regimens.

Conversely, statistically significant differences can be seen in Table 2, with only one patient being prescribed d4T in 2017 compared with 20 in 2016 (*p* < 0.0001). The number of patients changing from an EFV-based regimen to an LPV/r-based regimen had also increased from 1.6% to 8.3% (*p* = 0.021), implying that more patients were being changed from failing first-line regimens to second-line regimens.

The frequency of weights documented did not differ significantly from 2016 to 2017, but there was a statistically significant reduction in prescription errors as shown in Table 3. Incorrect dosing, as assessed by dosage according to weight and frequency as per the national guidelines,^20^ dropped from 51.1% to 35% (*p* = 0.0004) – whilst underdosing almost halved from 11.7% to 6.4% (*p* < 0.0001) from 2016 to 2017. The percentage of patients being prescribed a daily instead of twice-daily dosing of eligible ART also increased significantly from 18.1% to 44.2% (*p* < 0.0001).

As shown in Table 4, an additional 16% (*p* < 0.0001) of patients had their VL taken within schedule in 2017 compared with 2016. The average wait for a repeat VL testing after an initial VL of more than 1000 RNA copies/mL also decreased from 9.2 to 5.3 months (*p* = 0.0011). With regard to the primary aim of the study, the percentage of suppressed patients in 2017 also significantly increased from 55.3% to 67.4% (*p* = 0.0088).

## Discussion

The use of a QI approach with a clinical mentorship intervention has resulted in significant improvements to various process measures, including an overall improvement in viral suppression in paediatric patients.

There was a dramatic reduction in the amount of d4t prescriptions in accordance with the 2013 recommendation to switch children from d4t-containing regimens owing to its potentially severe metabolic toxicities.^29^ The number of patients changed from an EFV-based regimen to an LPV/r-based regimen increased, implying that more children were changed from a failing first-line regimen with a non-nucleoside reverse transcriptase inhibitors backbone to a second-line regimen with a protease inhibitor backbone. At baseline, more than half of patients had an incorrect dose prescribed in the last year, which improved to 35% at the second audit. Although this remains alarmingly high, it is in keeping with findings from a study in a similar rural setting^30^ and could be an indication of the complexity of paediatric prescribing.^31^ Underdosing significantly improved from 11.7% to 6.4%, whilst the number of daily dosing prescriptions for ABC/3TC combination more than doubled from 18.1% to 44.2%. Viral load monitoring and response to VLs improved, with 16% more patients having their VLs performed within schedule, and the average delay between repeating a VL testing after an initial high VL down from 9.2 to 5.3 months, which is still longer than the 2 months recommended by the national guidelines.^20^ The most significant of the clinical findings was an improvement in viral suppression from 55.3% to 67.4%. Despite the improvement, this is still far short of the 90% suppression goal.

A significant limitation of the study was the 12 months allowed for the intervention before its effect was assessed. We anticipate that repeated data collection after a further 12 months would have shown further improvement in viral suppression. This is due to the fact that there is a minimum of 8 months from when the initial high viral load of a failing first line regimen is taken, to when the initial viral load on a second line regimen is taken. A further limitation was the lack of standardisation across the clinics with regard to the implementation of the clinical mentorship. Confidence in dealing with paediatric HIV varied from doctor to doctor, as did enthusiasm for PHC. This led some doctors to taking more initiative than others. A general limitation of the QI process is that we are unable to prove causation. During 2016 and 2017, more data capturers were employed at the clinics, which could also have contributed to improved outcomes.

Recommendations for future QI cycles include interventions focussing on data management, specifically data capturers and their interaction or communication with clinicians. The TIER.Net system has functions that enable the flagging of patients where VLs are due or where consecutive VLs are more than 1000 RNA copies/mL. We hope to improve the use of TIER.Net, its data accuracy and the use of its functions. The employment of extra data capturers at the clinics during 2017 has made this a feasible option. Additional recommendations include interventions focussing on prescription error rates, specifically improved dosing by weight. One suggestion has been to develop a weight-based ART dosing wheel (similar to the pregnancy wheel used by midwives to calculate gestation).

## Conclusion

The QI approach to addressing poor viral suppression has proved to be beneficial at the Madwaleni feeder clinics. We anticipate that this positive exposure to QI will encourage the Madwaleni doctors and clinic nurses alike to make use of this approach in the future.

Twelve months of clinical mentorship appears to have had a positive impact on viral suppression and paediatric HIV care in general. The ongoing doctor support of PHC nurses in fulfilling their task-shifting role in this very rural context appears to be crucial to the programme’s success.

## References

[1] Donald Woods Foundation HIV and TB. Health care is a basic human right, regardless of economic or financial means [homepage on the Internet]. [cited 2020 Mar 14]. Available from: https://www.donaldwoodsfoundation.org/hiv-and-tb/

[2] Malan M Rural hospitals in terminal crisis. Mail & Guardian [homepage on the Internet]. 2012 [cited 2020 Mar 14]; Available from: https://mg.co.za/article/2012-09-07-00-rural-hospitals-in-terminal-crisis/

[3] Osler M, Hilderbrand K, Hennessey C, et al A three-tier framework for monitoring antiretroviral therapy in high HIV burden settings. J Int AIDS Soc. 2014;17(1):18908 10.7448/IAS.17.1.18908 24780511 PMC4005043

[4] United Nations Programme for HIV/AIDS UNAIDS Data 2018 [homepage on the Internet]. Geneva: UNAIDS; 2018 [cited 2019 Nov 10]. Available from: https://www.aidsdatahub.org/sites/default/files/publication/UNAIDS_Data_2018.pdf

[5] United Nations Programme for HIV/AIDS 90-90-90: An ambitious treatment target to help end the AIDS epidemic. Geneva: UNAIDS; 2014.

[6] South African National Department of Health Clinical mentorship manual for integrated services. Pretoria: Department of Health; 2011.

[7] Sanne I, Orrell C, Fox MP, et al Nurse versus doctor management of HIV-infected patients receiving antiretroviral therapy (CIPRA-SA): A randomised non-inferiority trial. Lancet. 2010;376(9734):33–40. 10.1016/S0140-6736(10)60894-X 20557927 PMC3145152

[8] Fairall L, Bachmann MO, Lombard C, et al Task shifting of antiretroviral treatment from doctors to primary-care nurses in South Africa (STRETCH): A pragmatic, parallel, cluster-randomised trial. Lancet. 2012;380(9845):889–898. 10.1016/S0140-6736(12)60730-2 22901955 PMC3442223

[9] Abrams EJ, Strasser S 90-90-90–Charting a steady course to end the paediatric HIV epidemic. J Int AIDS Soc. 2015;18(20296). 10.7448/IAS.18.7.20296 PMC4670839 26639119

[10] Boerma RS, Boender TS, Bussink AP, et al Suboptimal viral suppression rates among HIV-infected children in low- and middle-income countries: A meta-analysis. Clin Infect Dis. 2016;63(12):1645–1654. 10.1093/cid/ciw645 27660236

[11] Boender TS, Sigaloff KCE, McMahon JH, et al Long-term virological outcomes of first-line antiretroviral therapy for HIV-1 in low- and middle-income countries: A systematic review and meta-analysis. Clin Infect Dis. 2015;61(9):1453–1461. 10.1093/cid/civ556 26157050 PMC4599392

[12] Chouraya C, Ashburn K, Khumalo P, et al Association of antiretroviral drug regimen with viral suppression in HIV-positive children on antiretroviral therapy in Eswatini. Pediatr Infect Dis J. 2019;38(8):835–839. 10.1097/INF.0000000000002347 31033912

[13] Fatti G, Bock P, Grimwood A, Eley B Increased vulnerability of rural children on antiretroviral therapy attending public health facilities in South Africa: A retrospective cohort study. J Int AIDS Soc. 2010;13(1):46 10.1186/1758-2652-13-46 21108804 PMC3002304

[14] Gaede B, Versteeg M Chapter 9. The state of the right to health in rural South Africa In: South African Health Review 2011. Durban: Health Systems Trust; 2011 p. 99–106.

[15] Lilian RR, Mutasa B, Railton J, et al A 10-year cohort analysis of routine paediatric ART data in a rural South African setting. Epidemiol Infect. 2017;145(1):170–180. 10.1017/S0950268816001916 27609130 PMC5197927

[16] Varkey P, Reller MK, Resar RK Basics of quality improvement in health care. Mayo Clin Proc. 2007;82(6):735–739. 10.1016/S0025-6196(11)61194-4 17550754

[17] Davidoff F, Batalden P, Stevens D, Ogrinc G, Mooney S Publication guidelines for quality improvement studies in health care: Evolution of the SQUIRE project. J Gen Intern Med. 2008;23(12):2125–2130. 10.1007/s11606-008-0797-4 18830766 PMC2596523

[18] Hirschhorn LR, Ramaswamy R, Devnani M, Wandersman A, Simpson LA, Garcia-Elorrio E Research versus practice in quality improvement? Understanding how we can bridge the gap. Int J Qual Health Care. 2018;30(suppl_1):24–28. 10.1093/intqhc/mzy018 29447351 PMC5909640

[19] SUCCEED The Stellenbosch University collaborative capacity enhancement through engagement with districts [homepage on the Internet]. [cited 2019 Mar 3]. Available from: http://succeed.sun.ac.za/

[20] National Department of Health National consolidated guidelines for the prevention of mother-to-child transmission of HIV (PMTCT) and the management of HIV in children, adolescents and adults. Pretoria: Department of Health; 2015.

[21] Musiime V, Kasirye P, Naidoo-James B, et al Once- versus twice-daily abacavir and lamivudine in African children: The randomised controlled ARROW Trial. AIDS Lond Engl. 2016;30(11):1761–1770. 10.1097/QAD.0000000000001116 PMC6217915 27064996

[22] Massoud R, Askov K, Reinke J, et al A modern paradigm for improving healthcare quality Quality Assurance (QA) Project. Bethesda, MD: U.S. Agency for International Development; 2001.

[23] Green A, De Azevedo V, Patten G, Davies M-A, Ibeto M, Cox V Clinical mentorship of nurse initiated antiretroviral therapy in Khayelitsha, South Africa: A quality of care assessment. Sued O, editor PLoS One. 2014;9(6):e98389 10.1371/journal.pone.0098389 24887260 PMC4041881

[24] Workneh G, Scherzer L, Kirk B, et al Evaluation of the effectiveness of an outreach clinical mentoring programme in support of paediatric HIV care scale-up in Botswana. AIDS Care. 2013;25(1):11–19. 10.1080/09540121.2012.674096 22533352 PMC3426665

[25] Van Deventer C, Golden L, Du Plessis E, Lion-Cachet C Optimal management of children on antiretroviral therapy (ART) in primary care: A quality improvement project. South Afr Fam Pract. 2017;59(1):29–34. 10.1080/20786190.2016.1254928

[26] Jones M, Cameron D Evaluating 5 years’ NIMART mentoring in South Africa’s HIV treatment programme: Successes, challenges and future needs. S Afr Med J [serial online]. 2017 [cited 2020 Jun 25];107(10):839–842. Available from: https://www.ajol.info/index.php/samj/article/view/164929 10.7196/SAMJ.2017.v107i10.12392 29022525

[27] Visser CA, Wolvaardt JE, Cameron D, Marincowitz GJO Clinical mentoring to improve quality of care provided at three NIM-ART facilities: A mixed methods study. Afr J Prim Health Care Amp Fam Med. 2018;10(1):1–7. 10.4102/phcfm.v10i1.1579 PMC6018522 29943605

[28] World Health Organization Monitoring services, patients and programmes In: Operations manual for delivery of HIV prevention, care and treatment at primary health centres in high-prevalence, resource-constrained settings. Geneva: World Health Organization; 2008; p. 96–104. 26290930

[29] National Department of Health The South African antiretroviral treatment guidelines. Pretoria: Department of Health; 2013.

[30] Dakshina S, Olaru ID, Khan P, et al Evaluation of weight-based prescription of antiretroviral therapy in children. HIV Med. 2019;20(3):248–253. 10.1111/hiv.12702 30632659 PMC6590156

[31] Van der Zanden TM, Goedknegt L, De Hoog M, Mooij MG, De Wildt SN, Van der Sijs IH Development and implementation of a paediatric dosing calculator integrated in the Dutch Paediatric Formulary. Drugs Ther Perspect. 2020;36(6):253–262. 10.1007/s40267-020-00724-y

